# Surgical restoration of drop-hand syndrome with tendon transfer in patients injured in the Syrian civil war

**DOI:** 10.1186/s40779-019-0225-6

**Published:** 2019-11-19

**Authors:** Murat Ucak

**Affiliations:** Plastic and Reconstructive Aesthetic Surgery, Hatay Defne State Hospital, 31000 Antakya, Hatay Turkey

**Keywords:** Hand, Tendon transfer, Drop-hand syndrome, Syrian civil war

## Abstract

**Background:**

The radial nerve is one of the most common war-related injury sites due to penetrating cutting tool injuries or gunshot wounds, resulting in drop-hand syndrome. The aim of this study was to evaluate the outcomes of tendon transfer in patients with drop-hand syndrome who had been injured in the Syrian Civil War.

**Methods:**

This level-II, prospective, comparative study included 13 civilians injured in the Syrian Civil War 2015 and 2017. The palmaris longus tendon was used for transfer to the extensor pollicis longus for thumb extension. The pronator teres was transferred to the extensor carpi radialis brevis for wrist extension. The flexor carpi radialis was transferred to the extensor digiti communis for 2nd, 3rd, 4th, and 5th finger extension. All outcomes of thumb abduction and extension, wrist extension, wrist flexion, and finger extension were assessed.

**Results:**

There was a high level of radial nerve injury in all patients included in the study. The time from injury to treatment ranged from 1.5 months to 9 months. The mechanism of injury most commonly observed was a gunshot wound, which was observed in 8 patients (61.5%), followed by a penetrating cutting tool injury (*n* = 3; 23.1%) and humerus fracture (*n* = 2; 15.4%).

**Conclusions:**

In radial nerve injuries, successful results can be achieved with tendon transfer. All patients regained thumb abduction of up to approximately 60°. All the patients were able to bend the wrist, grip, and extend the fingers while in wrist flexion, neutral wrist and wrist extension positions. Although the reason for the radial injury varied, the postoperative outcomes were good for all patients, and the rehabilitation period progressed successfully in patients who underwent tendon transfer repair within 90 days of injury.

## Background

Throughout the world, civilians are suffering the adverse effects of wars. In Syria, severe injuries have been sustained by the civilian population [1]. It has been estimated in reports that 11.5% of the Syrian civilian population has been killed or injured since the crisis erupted in 2011 [2]. The number of wounded individuals increased to approximately 1.9 million in 2017 [3]. As Turkey is one of the neighboring countries of Syria, surgeons working in nearby cities such as Urfa, Gaziantep, Antakya, and Adana have performed many operations on Syrian patients [4].

It is well known that wars present different kinds of injuries that can lead to permanent injury, disability or limb loss [5, 6]. The radial nerve is one of most common war-related injury sites due to penetrating cutting tool injuries or gunshot wounds, resulting in drop-hand syndrome [7]. Although surgical treatment for this wound pattern is controversial among surgeons of the upper extremity, certain management principles are valid in all cases. Although the principles and clinical outcomes of different surgical treatments vary [8], all plastic surgeons have adapted their own approaches to improve postoperative outcomes and avoid any long-term morbidity risks [9–11].

Tendon transfer is applied following peripheral nerve injuries that present with delayed reinnervation of the related muscle, with a lack of function due to motor fibrosis [12]. In such cases, transferring tendons is the only chance for a patient to regain wrist or finger extension. Although different tendon options are available, surgeons prefer the use of specific tendon groups [13]. As a general reference, wrist extension restoration is employed with the transfer of the pronator teres (PNT) to the extensor carpi radialis brevis (eCRB) [14]. Palmaris longus (PML) is the best choice for restoration of thumb extension and thumb radial abduction [13, 15]. The repair approach for finger extension can be employed using the flexor digitorum superficialis (fDS), flexor carpi ulnaris (fCU), or flexor carpi radialis (fCR) [16].

To date, there have been no other reasonable alternatives to tendon transfers for radial neuron palsies following a severe injury such as that sustained in armed conflict. Although a few studies have been conducted in Turkey regarding war injuries from the Syrian Civil War, this is the first study regarding surgical restoration of drop-hand syndrome with tendon transfer in Syrian patients.

## Methods

This level-II, prospective, comparative study included civilians injured in the Syrian Civil War 2015 and 2017. All patients acknowledged and signed the informed patient consent form for the study after the completion of demographic data collection [17]. We obtained study approval from the institute where the study was conducted (Number: 2018–255-0002). A total of 13 patients were determined to have radial nerve palsy. Ternary tendon transfer surgery was performed on all of these injured Syrian patients. The choice of the appropriate surgical method was based on the complete evaluation of the patient, comprising a clinical evaluation (anamnesis, clinical and neurological examination), an electrophysiological evaluation (EMNG, SSEP), a radiological evaluation (X-ray, CT, MRI), and an intraoperative evaluation with the use of intraoperative monitoring, as well as on the treatment of symptoms (pain, motor, sensory or cosmetic deficits).

### Surgical technique for tendon transfer

**Fig. 1:**
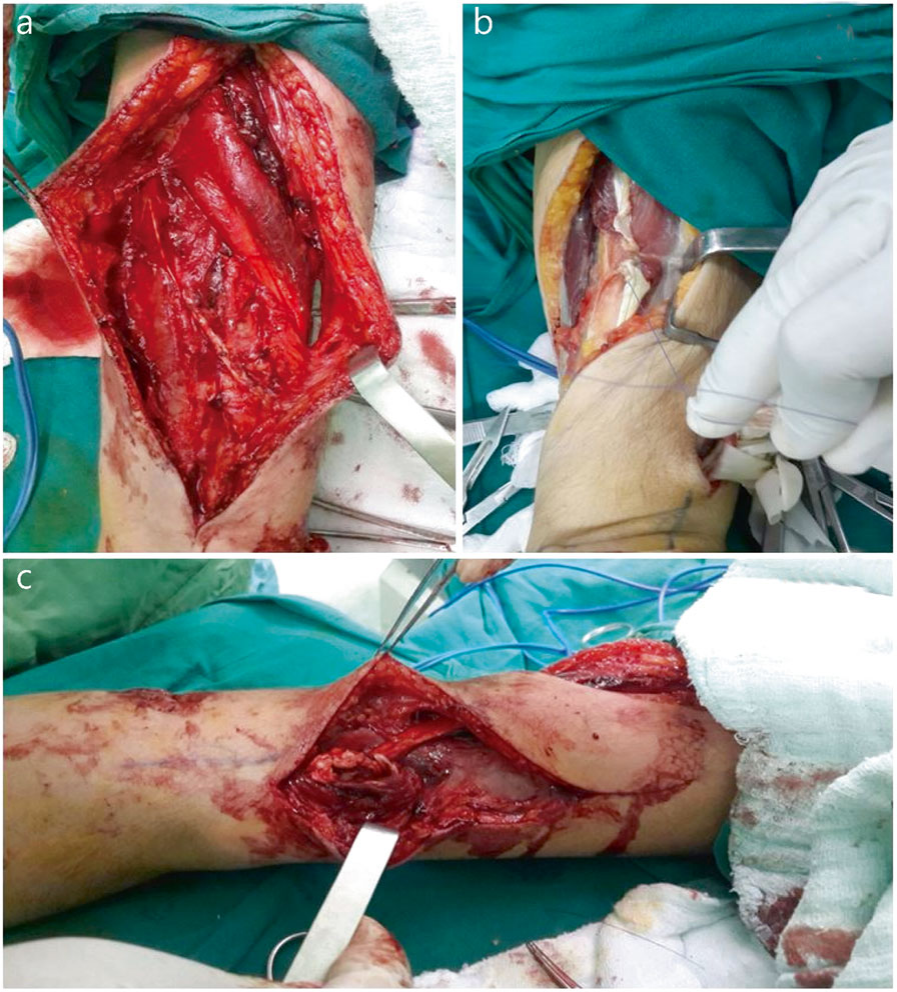
The palmaris longus tendon was used for transfer to the extensor pollicis longus for thumb extension, as shown in (**a**). The pronator teres was transferred to the extensor carpi radialis brevis for wrist extension, as shown in (**b**). The flexor carpi radialis was transferred to the extensor digiti communis for 2nd, 3rd, 4th, and 5th finger extension, as shown in (**c**)

All operations were performed under general anesthesia with tourniquet control. In the surgical tendon transfer technique, the palmaris longus tendon (PLT) was used for transfer to the ePL for thumb extension, as shown in Fig. 1a. The pronator teres was transferred to the eCRB for wrist extension, as shown in Fig. 1b. The flexor carpi radialis was transferred to the extensor digiti communis (eDC) for 2nd, 3rd, 4th, and 5th finger extension, as shown in Fig. 1c. All tendon repairs were performed “end-to-end” with polypropylene suture, and hands were immobilized after surgery.

The tendons were cut distally at the wrist and freed from the musculotendinous junction proximally after the tendons of the fCU and fCR were released via longitudinal forearm incisions. The tendons were tunneled along the radial and ulnar borders of the forearm subcutaneously. Under high tension, the tendon was sutured to the eCRB tendon with the wrist extended. The fCU was joined with the abductor pollicis longus (APL), extensor pollicis longus (ePL), eDC, and extensor indices proprius (eIP). After wrist extension under tension, the fCU was connected to the eDC and eIP, which are the finger extensor muscles. This approach was 15° short of full extension for the metacarpophalangeal joints. To provide full extension for the thumb, the ePL tendon was joined with the fCU tendon first, and then the APL tendon was sutured to the fCU tendon at maximum tension.

### Assessment of results

**Fig. 2:**
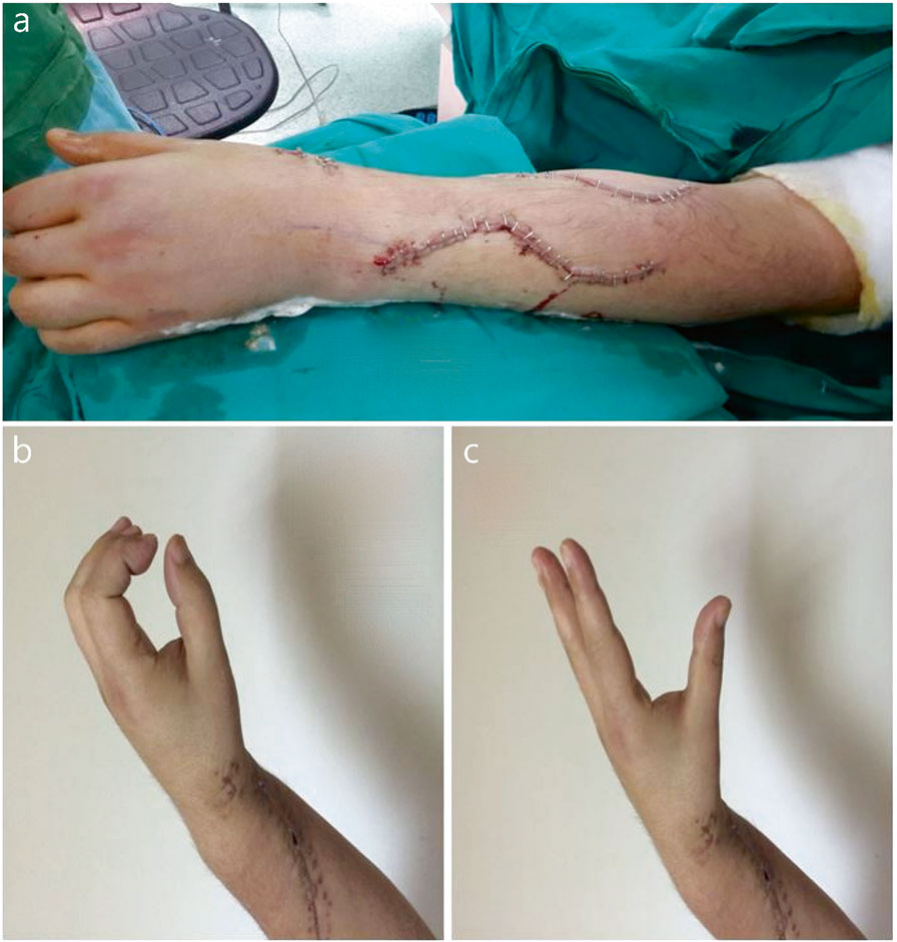
Patient images taken immediately following the operation (**a**) and 45 days postoperatively (**b-c**)

**Fig. 3:**
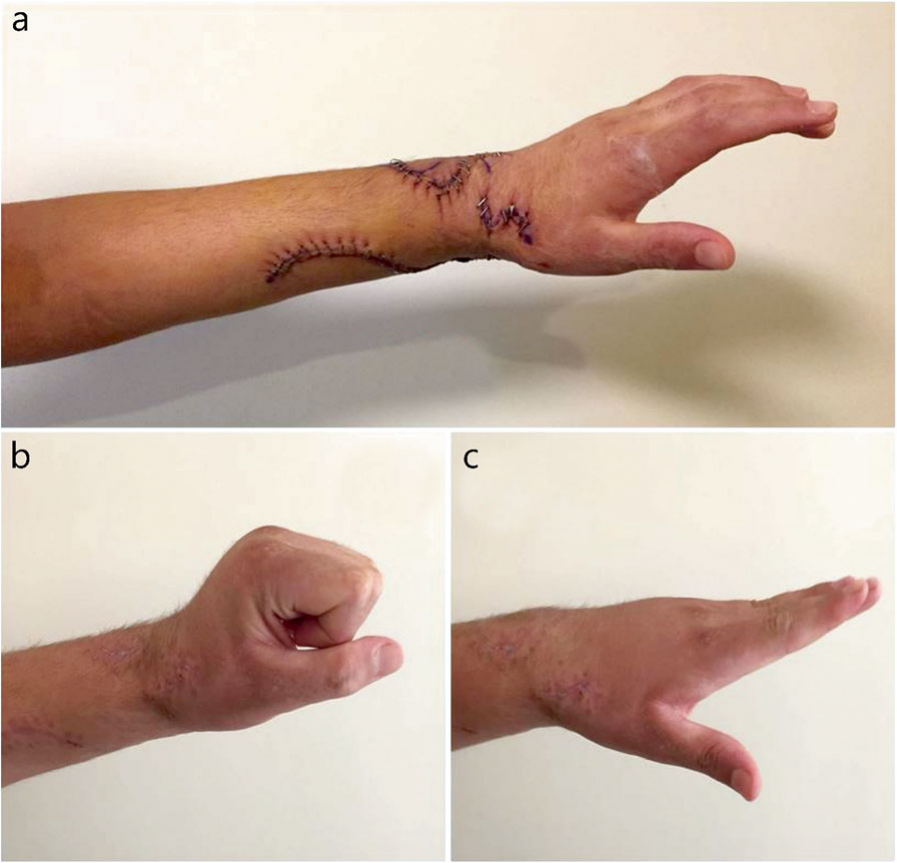
Patient images taken immediately following the operation (**a**) and 90 days postoperatively (**b-c**)

The overall result was rated as perfect, good, fair, or poor according to the Moussavi et al. [10] classification. The assessment criteria are shown in Table 1. Thumb abduction and extension, wrist extension, wrist flexion, and finger extension were assessed according to these ratings, and the ability of the patient to return to their preinjury occupations was determined as a percentage (Figs. 2-3).

**Table 1:** The surgical functional criteria for motion assessment

Movement	Perfect	Good	Fair	Poor
WE	0°-80°	0°	45° Extension restriction	70° Extension restriction
FE	0°-10°	0°	45° Extension restriction	90° Extension restriction
TM	80°- 99°	60°-80°	30°-59°	0°-29°
WF	Exact	0°-20°	0°	Dorsiflexed

*FE* Finger extension, *WE* Wrist extension, *WF* Wrist flexion, *TM* Thumb movement

### Statistical analysis

The statistical analysis of data was performed using SPSS 24.0 software (IBM Corp, New York, USA) to evaluate descriptive statistics such as mean and standard deviation. We did not have date to compare between groups.

## Results

The study included a total of 13 patients with drop-hand syndrome resulting from injuries sustained in the Syrian Civil War. The patients comprised 11 males and 2 females with a mean age of 30.6 ± 13.2 years. The most common mechanism of injury was a gunshot wound, which was observed in 8 patients (61.5%), followed by a penetrating cutting tool injury (*n* = 3; 23.1%) and humerus fracture (*n* = 2; 15.4%).

The patient occupations reported were mostly student and soldier. The operating time ranged from 45 min to 270 min. The ability to return to preinjury occupation was > 80% for most patients. The postoperative follow-up outcomes and demographic details of the patients are shown in Table 2.

**Table 2:** Detailed documentation of the patients and functional assessment outcomes

ID	Sex	Age (year)	Occupation	Injury	Time^*^(min)	FE	WE	WF	TM	Ability toreturn (%)
1	Female	66	Housewife	DCI	45	Good	Perfect	Good	Perfect	100
2	Male	37	Photographer	DCI	80	Perfect	Good	Good	Good	90
3	Male	15	Student	DCI	110	Good	Good	Good	Perfect	90
4	Male	48	Merchant	GW	95	Good	Good	Good	Good	80
5	Male	22	Waiter	HF	90	Good	Good	Good	Good	80
6	Male	17	Student	GW	120	Good	Good	Good	Good	85
7	Female	28	Housewife	GW	75	Good	Good	Perfect	Good	90
8	Male	28	Soldier	GW	160	Good	Fair	Good	Good	70
9	Male	29	Pharmacist	GW	100	Good	Good	Good	Good	80
10	Male	31	Teacher	HF	95	Good	Good	Good	Good	80
11	Male	21	Soldier	GW	120	Good	Good	Fair	Good	70
12	Male	23	Soldier	GW	143	Good	Good	Good	Good	70
13	Male	33	Worker	GW	270	Good	Fair	Poor	Good	50

Time*: Time of tendon transfer after injury; *FE* Finger extension, *WE* Wrist extension, *WF* Wrist flexion, *TM* Thumb movement, *DCI* Drill cutter injury, *GW* Gunshot wound, *HF* Humerus fracture

## Discussion

It is well known by surgeons that tendon transfer is of critical importance in the restoration of lack of hand function and decreased limb performance [10, 18]. This is because once a nerve repair application has failed, better results can be obtained via tendon transfer in patients with radial nerve palsy. In particular, if there is a low level of improvement in radial nerve injuries within a one-year period, tendon transfer should be recommended for the treatment of such injuries [14, 19, 20]. Although many surgeons prefer different tendon transfer approaches to obtain the best treatment outcomes, including restoration of the thumb, finger extension, and wrist extension, there are points of disagreement on the best tendon transfer approach in patients with radial nerve palsy [21, 22]. In this paper, the results were evaluated for tendon transfer applied to 13 Syrian patients with drop-hand syndrome, and promising outcomes were obtained.

The use of heavy weapons resulting in serious kinetic energy injuries has been reported as a routine consequence of the conflict in Syria [23]. The transfer time of patients in these circumstances can be longer than what is normally expected, and infections may be observed at higher rates in these cases [5]. Civilian surgeons located closest to the war are ideally obliged to treat injured civilians [6]. As Turkey is a country neighboring Syria, surgeons in cities close to the border treat wounded civilians from the ongoing civil war [4]. Among injured Syrians, limb injuries are one of the most commonly encountered pathologies. As a result of limb injuries, severe radial nerve damage has been reported in these patients. In the literature, most reports of drop-hand syndrome have occurred during iatrogenic humeral fracture repair [24–26]. However, in the current study, most of the injuries were due to penetrating cutting tool injuries, while only two cases occurred during iatrogenic humeral fracture repair.

The best approach for radial nerve injuries, especially in the setting of a closed humeral shaft fracture, continues to be controversial among plastic surgeons. Transfer of the pronator teres to the extensor carpi radialis brevis is the most reliable approach to restore wrist extension [15]. However, there is sometimes no possibility of radial nerve recovery, and it is necessary to perform the transfer using the end-to-end approach, in which the extensor carpi radialis brevis tendon is cut and sutured to the endpoint of the pronator teres tendon. This method results in a flat tension direction and more efficient transfer. If the radial nerve has already been repaired and it is anticipated that the extensor carpi radialis brevis will recover in the future, the pronator teres tendon should be transferred using the end-to-end method, suturing the tendon adjacent to the intact extensor carpi radialis brevis tendon [27–29]. When this application is performed early, the nerves will function as an internal splint to correct the wrist while healing. Consequently, the extensor carpal radialis brevis is kept in good condition, and wrist extension will be restored when motor function is regained [15]. In the surgical tendon transfer technique used in the current study, the pronator teres was transferred to the extensor carpi radialis brevis for wrist extension, and the flexor carpi radialis was transferred to the extensor digiti communis to provide 2nd, 3rd, 4th, and 5th finger extension.

Thumb extension is important for achieving high-level hand functioning. The combined extension movement of the thumb and index finger must be simultaneous. This movement facilitates correct manipulation and allows for unique motion [14, 29]. Transfer of the palmaris longus to obtain motion may result in an extension of the joints between the fingers as well as thumb abduction in the radial direction. Several different tendons may be transferred to the extensor pollicis longus. In the literature, the most frequently transferred tendons are the palmaris longus or flexor digitorum superficialis of the ring finger [12, 13, 20]. Similarly, the palmaris longus tendon was transferred to the extender pollicis longus for thumb extension in the current study. The follow-up results of these patients were satisfactory, as their condition demanded.

Transferring the flexor digitorum superficialis, flexor carpi ulnaris, or flexor carpi radialis tendons to the extensor digitorum communis may be a solution to restore extension of the metacarpophalangeal joints of the fingers. In a study by Yavari et al. [30], a single tendon transfer from the flexor carpi ulnaris to the common extensor digitorum and extensor pollicis longus was performed on 30 subjects. Ternary tendon transfer from the pronator teres to the extensor carpi radialis brevis, from the flexor carpi ulnaris to the common extensor digitorum and from the palmaris longus to the extensor pollicis longus was applied to another 17 patients. There was no significant difference between the results of single tendon and ternary tendon transfer surgery after 1 month of postoperative splinting and physiotherapy. In the current study, ternary tendon transfer was used rather than single tendon transfer, so there was no comparison. The majority of the Syrian patients regained their preinjury occupational ability. We believe that these patients fought as guerrillas with high motivation and that there was no regular army. In fact, some of the patients who were treated went to war again and were injured again. The most important point of the current study was that with the application of immediate tendon transfer surgery, a considerable amount of wrist extension was regained.

There were some limitations to this study. Long-term follow-up of most patients was not possible because they were from Syria and had no long-term address; thus, they were lost after discharge.

## Conclusion

In conclusion, the present surgical applications to patients from the Syrian War showed that all patients regained thumb abduction up to approximately 60°. All patients regained the ability to bend the wrist and to grip, and they could extend the fingers in wrist flexion, neutral wrist, and wrist extension positions. To the best of our knowledge, this is the first study to have presented results on radial nerve injuries that occurred in the Syrian Civil War. Radial nerve injuries can vary in terms of whether they were caused by armed conflict or by accident. In the current study, injuries due to gunshot or cutting tool wounds were more frequently observed than injuries due to humerus fracture, which are more common in accidental injuries. In patients with radial nerve injury due to war, successful results can be obtained with tendon transfer surgery. Despite the varying causes of radial injury, the postoperative outcomes were good, and the rehabilitation period progressed successfully in patients who underwent tendon repair within 90 days of the injury.

## Data Availability

Not applicable.

## Abbreviations

APL: Abductor pollicis longus
eCRB: Extensor carpi radialis brevis
eDC: Extensor digiti communis
eIP: Extensor indices proprius
ePL: Extensor pollicis longus
fCR: Flexor carpi radialis
fCU: Flexor carpi ulnaris
fDS: Flexor digitorum superficialis
PLT: Palmaris longus tendon
PML: Palmaris longus
PNT: Pronator teres
